# Stromal Regulation of Tumor Perineural Invasion: A Multicellular and Neuro‐Ecological Perspective

**DOI:** 10.1002/advs.76248

**Published:** 2026-06-28

**Authors:** Xiaoyang Lin, Yingqiao Liu, Haitao Lin, Kang Feng, Xiqiang Liu

**Affiliations:** ^1^ Department of Oral and Maxillofacial Surgery Nanfang Hospital Southern Medical University Guangzhou China

**Keywords:** cancer‐associated fibroblasts, perineural invasion, schwann cells, stromal regulation, tumor microenvironment

## Abstract

Perineural invasion (PNI) is an aggressive driver of tumor progression, therapeutic resistance, and poor prognosis across diverse malignancies. Traditionally viewed as a tumor cell‐autonomous process, PNI is increasingly recognized as a dynamic phenomenon shaped by the tumor–nerve–stromal ecosystem. This review synthesizes recent advances to reframe neural invasion as a coordinated, multicellular process governed by reciprocal interactions within this niche. We delineate how stromal and immune components, including cancer‐associated fibroblasts, Schwann cells, immune cells, and the extracellular matrix, facilitate PNI through structural remodeling and biochemical signaling within the tumor–nerve interface. These coordinated interactions drive nerve reorganization and local immune reprogramming, collectively establishing a permissive niche that supports tumor spread along neural routes. By integrating these interconnected mechanisms into a neuro‐ecological framework, we clarify how networked tumor–stromal–neural remodeling underlies neural dissemination. This perspective identifies therapeutic vulnerabilities within the neural microenvironment and suggests that disrupting bidirectional crosstalk between tumor cells and their stromal partners may offer strategies to limit neural spread and improve clinical outcomes.

## Graphical abstract

This review reframes perineural invasion (PNI) as a dynamic, multicellular process within the tumor–nerve–stromal ecosystem. The authors delineate how cancer‐associated fibroblasts, Schwann cells, and immune cells remodel the neural niche to facilitate tumor spread. By integrating these interactions into a neuro‐ecological framework, this work identifies key therapeutic vulnerabilities to limit neural dissemination and improve clinical outcomes.

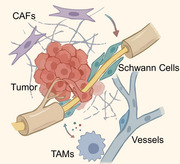

## 1 Background

Perineural invasion (PNI) refers to the presence of tumor cells infiltrating any of the three layers of the nerve sheath—the epineurium, perineurium, or endoneurium—or encircling ≥33% of the nerve circumference [1]. Although its incidence varies considerably across cancer types, ranging from approximately 80%–100% in pancreatic ductal adenocarcinoma (PDAC) to 20%–80% in head and neck cancers, PNI constitutes a prominent and clinically significant pathological feature across diverse solid malignancies [2]. Clinically, PNI is not merely a histological finding but is recognized as an independent adverse prognostic factor in multiple solid tumors and is closely associated with tumor progression, pain, therapeutic resistance, local recurrence, distant metastasis, and reduced survival [1, 2, 3, 4].

Early theories posited that PNI was the result of the mechanical extension of cancer cells along the “path of least resistance” [1]. However, this mechanical paradigm cannot adequately account for the active, tumor‐directed neurite outgrowth, the alterations in the local immune microenvironment, and the pronounced neurotropism exhibited by certain cancers. Subsequent studies demonstrated that nerves are not passive victims in PNI; rather, under tumor‐derived signals, they are actively “recruited” or “remodeled” [5, 6]. This bidirectional crosstalk is driven by tumor‐secreted neurotrophic factors, such as nerve growth factor (NGF) and brain‐derived neurotrophic factor (BDNF), that induce axonal extension toward the tumor bed, while nerves reciprocally facilitate tumor migration and proliferation through paracrine signaling, structural guidance, and even mitochondria transfer [7, 8, 9].

**FIGURE 1:**
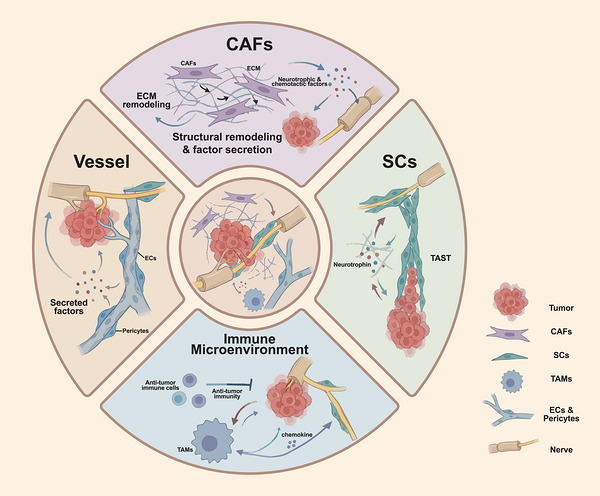
The TME Landscape and Its Contributions to PNI. The tumor stroma plays a central role in shaping a permissive niche for PNI. (A) Distinct stromal components—including CAFs, SCs, vascular cells, immune cells, and the ECM—cooperate to promote neural remodeling and tumor neurotropism. (B) CAFs remodel the ECM and secrete neurotrophic and chemotactic factors. (C) Tumor‐activated SCs guide tumor cell migration along nerves via direct contact and secretion of neurotrophic factors. (D) Immune cells, especially tumor‐associated macrophages, regulate inflammation and neural barrier integrity. (E) Vascular components provide paracrine signals at the tumor–nerve interface.

With the deepening understanding of neural anatomy and the tumor microenvironment (TME), increasing evidence indicates that within the specialized microenvironment of neural invasion, stromal components, including cancer‐associated fibroblasts (CAFs), immune cells, Schwann cells (SCs), and others, are both shaped by neural signals and actively involved in promoting neural remodeling and tumor migration toward nerves [7, 10]. They achieve this by secreting neurotrophic factors, chemokines, and cell adhesion molecules, and by remodeling the extracellular matrix (ECM). This “third‐party” component acts as both a scaffold and an amplifier, expanding the concept of PNI from a localized cell–cell interaction to a complex multicellular ecosystem and tripartite network involving tumor, nerves, and stroma (Figure 1).

In this review, we integrate emerging evidence to construct a multicellular, neuro‐ecological framework of PNI. By dissecting how stromal populations orchestrate this process, this review moves beyond the traditional bipartite tumor–nerve model and highlights stromal nodes as potential therapeutic targets. Notably, while the central focus of this review is placed on stromal regulation of PNI, neural‐intrinsic signaling pathways, such as adrenergic signaling and sensory pathways, also represent important modulators of tumor progression. Adrenergic signaling may amplify stromal NGF production, immune suppression, and neural remodeling [5]. Also, sensory nerves and neurotransmitter‐mediated signaling may influence pain, neurogenic inflammation, and tumor‐associated neural plasticity [11]. These pathways should therefore not be viewed as separate from stromal regulation, but rather as convergent neural inputs that may modulate PNI initiation, progression, and symptom burden.

## 2 The Tumor Stroma as a Perineural Niche: Composition and Ecological Perspectives

The tumor stroma constitutes a complex ecosystem comprising cancer‐associated nonmalignant cells and ECM components. It provides the environmental foundation that supports tumor growth, invasion, and neural innervation, and represents the structural and functional core of the TME, comprising both cellular and non‐cellular components. Traditionally, cellular components of the tumor stroma have been classified to include CAFs, vascular endothelial cells, pericytes, and immune cells. With the integration of multi‐omics approaches, including single‐cell sequencing and spatial transcriptomics, and increasing resolution of the perineural niche, additional neural‐associated cell types (such as SCs), as well as organ‐specific stromal cells (for example, pancreatic stellate cells in the PDAC microenvironment), have also been recognized as key constituents [12, 13]. The non‐cellular compartment is primarily composed of ECM and various soluble mediators and metabolites. These components not only form the “habitat” of cancer cells but also serve as critical bridges linking cancer to the broader organism [14] (Figure 1).

The tumor stroma not only provides structural support but also shapes tumor–nerve interactions. Increasing quantitative evidence suggests strong correlations between stromal proportion, stromal composition, and the incidence of PNI [14]. Transcriptomic analyses indicate that reactive stroma upregulates neural development‐related gene programs [15], correlating with PNI incidence and severity across multiple tumor types [16, 17, 18]. Crucially, the impact of the stroma on PNI extends beyond abundance to histological composition; for example, fibroblast‐rich stromal architectures, such as fibromyxoid responses, are linked to increased neural invasion and poor prognosis in vulvar squamous cell carcinoma [19]. Collectively, these studies establish a strong association between stromal abundance and composition and tumor neural invasion at both macroscopic and microscopic levels.

Rather than being static markers, these molecular and histological associations reflect a functional transformation of the neural landscape. At the morphological level, this stroma‐driven interaction is characterized by dynamic spatial shifts in nerve density and remodeling of neural architecture. In many solid malignancies, such as prostate and breast cancers, axonal networks are preferentially enriched at the invasive front, where tumor‐ and stroma‐driven axonogenesis and sprouting expand the physical interface for tumor–nerve interactions [20]. Increased nerve density and neural remodeling may mark earlier or parallel phases of perineural niche formation, whereas histologically confirmed PNI more directly reflects tumor engagement with nerve‐associated structures. Integrating these morphological features with stromal and molecular analyses may therefore clarify how the perineural niche forms and evolves.

While existing studies have extensively mapped pairwise tumor–nerve interactions, understanding PNI fully requires integrating these distinct stromal contributions into a broader systems‐level perspective. To synthesize this complexity, we conceptualize the perineural microenvironment through a “neuro‐ecological” framework. This perspective frames PNI as an evolving multicellular ecosystem shaped by interconnected processes involving stromal remodeling, reciprocal signaling interactions, spatial organization, and temporal progression.

Within this framework, the stromal components can be viewed as key contributors that together influence the structure and signaling dynamics of the perineural niche. In this context, dissecting the specific contributions and mechanisms of individual stromal components, as well as their potential interactions, remains essential for understanding the stromal basis of PNI. We next synthesize emerging evidence on CAFs, SCs, immune cells, and other stromal constituents as active regulators of neural invasion.

## 3 The Impact of CAFs on PNI

As a major mode of invasive dissemination in many solid tumors, PNI is highly shaped by the TME. Among stromal populations, CAFs act as central organizers regulating multiple stages of PNI by restructuring the ECM to create permissive invasion paths and serving as signaling hubs that coordinate tumor–nerve–stroma interactions.

### 3.1 CAFs Activation and ECM Remodeling Establish the Structural Basis of the Perineural Niche

CAFs and the ECM together constitute both the structural basis and the functional supporting framework of the perineural niche within the TME. Fibroblasts, largely derived from mesenchymal stem cells, can be activated into CAFs by multiple cues in tumors, including TGF‐β, inflammatory, and mechanotransductive signaling pathways [21, 22]. Once activated, CAFs serve as the principal drivers of ECM deposition and remodeling. CAFs modulate integrin‐dependent adhesion and cytoskeletal organization [23], enhance ECM crosslinking and stiffness [24], and promote fiber alignment, thereby generating anisotropic tracks that facilitate directional tumor invasion and metastatic colonization [25, 26, 27]. The perineurium, which serves as the natural interface between tumors and nerves, is likewise derived from fibroblast lineages [28]. Moreover, the endoneurium, which represents the final barrier encountered as cancer cells infiltrate deeper into the nerve, is likewise enriched in fibroblasts [13]. Thus, CAF activation may be considered an important contributor to structural remodeling of the perineural niche.

### 3.2 CAF‐Specific Roles and Mechanisms in PNI

Emerging evidence positions CAFs as central architects of the perineural niche. Beyond the general pro‐tumorigenic functions, CAFs actively drive PNI through two main dimensions: structural remodeling and paracrine signaling networks.

#### 3.2.1 Structural Interactions between CAFs and the Perineural Niche and Their Role in PNI Initiation

High stromal expression of CAFs is significantly associated with PNI [29]. Importantly, CAF subtypes contribute unequally to this process. In a study integrating single‐cell transcriptomics with spatial transcriptomics in PDAC from 25 patients, myofibroblastic CAFs(myCAFs) and tumor‐like CAFs(tCAFs) were significantly enriched in tissues with high PNI, whereas progenitor‐like CAFs were preferentially distributed in regions with low neural invasion [13]. Consistently, α‐SMA–expressing myofibroblasts correlate with poor outcomes in head and neck squamous cell carcinoma (HNSCC). Galectin‐1–driven CAF activation further promotes tumor progression through α‐SMA induction and CCL2 secretion [30, 31].

Within the perineural niche, CAFs show lineage and spatial overlap with perineurial fibroblasts, suggesting a direct role in compromising perineurial barrier integrity [32]. Supporting this concept, perineural CAFs selectively express matrix metalloproteinase‐2 (MMP‐2), which facilitates ECM degradation and loosening of perineural structures, whereas CAFs from non‐PNI regions lack MMP‐2 expression [33]. This mechanism is further corroborated in colorectal cancer, where single‐cell transcriptomic analyses identified a highly enriched subset of MMP2^+^ myCAFs specifically within PNI‐positive tumors. Driven by the core regulatory gene PRRX2, these MMP2^+^ myCAFs actively disrupt the perineural barrier through extensive ECM remodeling, directly facilitating the perineural infiltration of CRC cells [34].

In addition to secreted proteases, CAF‐associated membrane proteins play critical roles during PNI. Fibroblast activation protein (FAP) has been implicated in enhancing cancer‐cell invasiveness, and its elevated expression correlates with PNI and unfavorable prognosis in diverse tumor contexts, such as PDAC and cutaneous squamous cell carcinoma (cSCC) [35, 36]. During perineural invasion, stromal components establish functional adhesive interfaces between tumor cells and nerves. Integrin α5 (ITGA5) expressed by tumor cells can bind to fibronectin 1 (FN1) in the ECM, thereby strengthening tumor cell adhesion to neural structures and promoting invasive behavior [37]. These findings highlight that the ECM is not merely a structural scaffold but actively drives tumor neurotropism through integrin‐dependent signaling pathways. Furthermore, membrane adhesion molecules expressed by CAFs, such as Nectin‐1, may facilitate direct contact and adhesion between tumor cells and neuronal processes, reinforcing cancer–nerve interactions and accelerating PNI progression [38].

Collectively, these lines of evidence indicate that CAFs establish a permissive histological and biophysical microenvironment for PNI by reshaping the perineural ECM, weakening structural barriers, and reinforcing adhesive interactions between tumor cells and neural elements.

#### 3.2.2 CAFs Orchestrate Neurotropism and Neural Remodeling via Paracrine Signaling Networks

Beyond physical barrier disruption and enhanced motility, CAFs orchestrate PNI through distinct neuro‐signaling interactions. In colorectal cancer, CAFs drive neural invasion via a β2‐adrenergic/NGF feedforward loop, enhancing sympathetic innervation and norepinephrine (NE) accumulation through PKA‐CREB and MAPK signaling [39]. Pharmacologic inhibition of β2‐adrenergic, PKA, or MEK/Trk signaling suppresses PNI [39]. This mechanism parallels the bidirectional signaling observed in adult bone marrow, where LepR^+^ stromal cells secrete NGF to maintain nerve fibers, while nerve fibers activate stromal signaling to promote regeneration [40]. In prostate cancer, CAFs activate YAP1/TEAD1 signaling in tumor cells to induce NGF expression, which in turn stimulates neuronal CCL2 release. Activation of the CCL2/CCR2 axis promotes epithelial–mesenchymal transition (EMT) and neural tropism, accelerating PNI [41]. In parallel, CAFs also serve as a direct and predominant source of NGF in the prostate microenvironment, establishing a reciprocal NGF/TrkA loop that drives EMT and facilitates tumor cell migration across neuronal layers [42]. Consistently, co‐culture studies demonstrate that fibroblast‐like stromal cells promote the growth of prostate cancer cells, stimulate neurite extension, and facilitate their encasement and invasion of nerves, supporting a CAF‐driven mechanism of neural invasion [43]. Similarly, in cholangiocarcinoma (CCA), CAFs construct a pro‐migratory paracrine milieu by secreting a robust network of inflammatory cytokines, including MCP‐1, CXCL‐1, IL‐6, and IL‐8, which directly enhances tumor cell motility and activates downstream oncogenic kinase pathways to drive invasion—a signaling crosstalk uniquely susceptible to targeted kinase inhibition [44]. In addition to protein‐based soluble signaling, CAF‐derived extracellular vesicles (EVs) can also facilitate PNI, expanding the modalities through which stromal cells regulate neural invasion [45].

Interestingly, CAF‐mediated metabolic reprogramming contributes to PNI, extending the spectrum of paracrine regulation beyond canonical protein signaling. In PDAC, upregulation of glycolysis in CAFs leads to lactate accumulation in the TME. This metabolic shift drives epigenetic modifications, specifically lactate‐mediated histone lactylation in cancer cells, which activate the transcription of PNI‐associated genes (such as L1 cell adhesion molecule (L1CAM) and slit guidance ligand 1 (SLIT1)) and fuel neural invasion [46].

#### 3.2.3 Inter‐stromal Crosstalk: CAF–Schwann Cell Coupling as a Niche Amplifier

In addition to their independent pro‐invasive functions, CAFs facilitate a reciprocal feedback loop with SCs to synergistically amplify the neuro‐stromal niche.

On one hand, CAFs actively modulate the functional state and plastic phenotypes of SCs. For instance, single‐cell and spatial transcriptomic analyses additionally reveal enrichment of TGF‐β–expressing myofibroblastic CAFs in PNI‐high regions, where they induce a pro‐invasive TGFBI^+^ SC phenotype [13]. Earlier studies indicated that CAF‐derived SLIT2 and LIF promote SC migration, differentiation, and neural plasticity via ROBO and JAK/STAT signaling [47, 48]. However, Zhang et al. (2025) demonstrated that, in OSCC, CAF‐derived LIF, which is significantly upregulated in metastatic cases, can drive SC inactivation rather than repair, thereby significantly exacerbating nerve injury and promoting overall PNI progression. This effect of SCs is characterized by reduced production of neurotrophic factors and concurrent demyelination, mechanistically linked to the activation of endoplasmic reticulum stress and the attenuation of MAPK signaling within SCs [49].

On the other hand, SCs reciprocally regulate the CAF compartment to further remodel the neuro‐stromal environment. SCs have been shown to secrete IL‐1α, which facilitates the polarization of CAFs toward an inflammatory phenotype (iCAFs). This SC‐mediated fibroblast reprogramming, combined with SC‐derived Midkine signaling that enhances tumor malignancy, creates a self‐reinforcing signaling network [50].

Collectively, this bidirectional CAF–SC coupling functions as a niche amplifier, where their mutually reinforced dysregulation interferes with neural homeostasis and creates a permissive environment for tumor expansion and PNI.

This pro‐invasive repertoire extends to tissue‐specific fibroblast‐like stromal subsets, most notably pancreatic stellate cells (PSCs) in PDAC. Functioning as the primary CAF precursors, PSCs mirror these mechanisms by upregulating NGF and MMP‐9 via the HGF/c‐Met/mTOR axis [51, 52] and driving PNI through Sonic Hedgehog signaling [53]. Additionally, PSC‐derived matricellular proteins, such as tenascin C, further enhance the bidirectional tropism between cancer cells and nerves [54].

**FIGURE 2:**
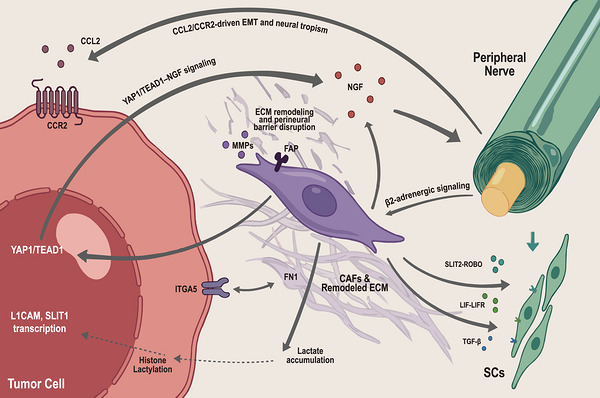
CAFs orchestrate PNI through coordinated structural remodeling and paracrine signaling networks. Structural remodeling: CAFs secrete proteases (MMPs, FAP) to remodel the ECM, compromising the perineural integrity and creating permissive physical conduits for invasion. Paracrine signaling networks: CAF‐driven signaling networks activate YAP1/TEAD1 programs in tumor cells to enhance NGF production and engage the CCL2/CCR2 axis, while CAF‐derived cues (SLIT2, LIF, and TGF‐β) modulate Schwann cell migration and phenotypic plasticity, collectively reinforcing neural remodeling. Complementing these signaling events, metabolic crosstalk via enhanced glycolysis and lactate accumulation in CAFs promotes the transcription of PNI‐associated genes (e.g., L1CAM, SLIT1) in tumor cells.

In summary, CAFs function as the multidimensional architects of the perineural niche. By orchestrating a multidirectional signaling network along the tumor‐nerve axis and simultaneously remodeling the physical ECM barrier, they drive invasion through synergistic structural and molecular mechanisms (Figure 2). However, current mechanistic insights are largely derived from pancreatic, prostate, and colorectal cancers; the role of CAFs in PNI within other malignancies remains underexplored. Crucially, the functional heterogeneity of CAF subsets suggests they may play distinct, and potentially opposing, roles in the PNI process. Future integration of single‐cell and spatial omics is essential to deconvolute these subpopulation‐specific mechanisms, potentially identifying novel therapeutic targets to intercept neural invasion and improve patient prognosis.

## 4 The Impact of SCs on PNI

As integral components of both the tumor stroma and the peripheral nervous system (PNS), SCs are key drivers of the onset and progression of PNI [55]. Histological examination reveals distinct co‐localization between GFAP^+^ SCs and cancer cells across diverse solid malignancies [56]. Historically, this proximity was interpreted as a passive anatomical juxtaposition, where SCs were viewed merely as structural bystanders or incidental victims of tumor‐induced damage. However, this paradigm has been fundamentally overturned. Rather than maintaining a quiescent state, SCs are reprogrammed at the pre‐invasive stage to facilitate PNI, utilizing intrinsic injury‐response mechanisms to guide cancer cell migration [55, 57, 58].

### 4.1 The Emergence of Tumor‐Activated Schwann Cells: Hijacking the Neural Repair Program

Under physiological conditions, mature SCs exhibit substantial plasticity. Upon nerve injury, SCs undergo c‐Jun–driven dedifferentiation [59], downregulating myelination genes (e.g., EGR2) and adopting a repair phenotype characterized by high expression of SOX2, p75NTR, and GFAP [60, 61]. Functionally, these repair Schwann cells (rSCs) execute a coordinated regeneration program: they secrete neurotrophic factors (e.g., Glial cell line‐derived neurotrophic factor(GDNF) and BDNF), align to form “Bands of Büngner” that serve as physical guides for regenerating axons, and modulate the immune microenvironment by recruiting macrophages to facilitate debris clearance [60]. Crucially, this intrinsic repair program provides a framework that is later co‐opted during tumorigenesis.

Consistent with this notion, tumor‐activated Schwann cells (TASCs) exhibit hallmark features of rSCs, including robust upregulation of c‐Jun and GFAP [37, 56, 58, 62], suggesting that tumor‐induced SC activation hijacks core elements of the intrinsic injury‐response program.

Most intuitively, physical invasion by tumor cells directly disrupts neural architecture, thereby triggering a local nerve injury response that indirectly activates SC demyelination and reparative reprogramming. Baruch et al. demonstrated that cancer cells cause myelin degradation and axonal injury through direct contact, resulting in local neuroinflammation [63]. Concurrently, studies in melanoma have revealed that physical compression and neuronal disruption during tumor expansion passively elicit a Wallerian‐like degeneration response. This mechanically and structurally driven injury forces mature SCs to downregulate myelination genes (e.g., Sox10, Egr2) and transdifferentiate into a dedifferentiated, “repair‐like” state [64]. Although SC dynamics were not explicitly delineated, the disruption of myelinating architecture physiologically implies SC dedifferentiation and transition to a repair phenotype, contributing to post‐injury neural remodeling. Consequently, this injury‐repair cascade is likely exploited by cancer cells to engage SCs in the pathological remodeling of the tumor‐neural microenvironment.

**FIGURE 3:**
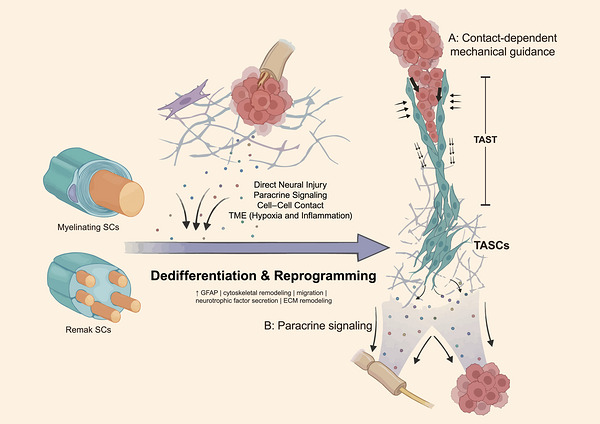
Activation of SCs into TASCs and their pro‐PNI functions. Under tumor‐associated microenvironmental cues, including neural injury, paracrine signaling, cell–cell contact, hypoxia, and inflammation, myelinating and Remak SCs undergo dedifferentiation and reprogramming into TASCs. This transition is accompanied by cytoskeletal remodeling, enhanced migration, neurotrophic factor secretion, and extracellular matrix remodeling. Activated TASCs promote perineural invasion through (A) contact‐dependent mechanical guidance, forming permissive tracks that physically guide tumor cell migration along nerves, and (B) paracrine signaling, whereby secreted neurotrophic and chemotactic factors facilitate tumor cell survival and nerve‐oriented invasion.

Moreover, multiple studies have revealed that tumors can actively activate SCs even prior to overt neural invasion via direct paracrine signaling, physical contact, or modulation of the TME. In PDAC, tumor cells induce c‐Jun‐dependent transcriptional reprogramming in SCs through either direct paracrine signaling or cell‐cell contact [58]. Cancer‐derived NGF binds to p75NTR (and potentially TrkA at high doses) on SCs, driving them into a highly migratory, activated state with specific tropism toward cancer cells [57, 65]. Subsequent studies in the complex TME of PDAC suggest SC activation is driven by synergistic cues that partially recapitulate the repair program following nerve injury [62]. Hypoxia, tumor‐associated inflammation, and cancer cell‐derived factors, particularly IL‐6, collectively form a potent SC activation network [62]. This active induction extends beyond pancreatic models; melanoma‐derived paracrine factors independently activate MAPK/Erk and Akt signaling pathways in SCs, driving the upregulation of immaturity and repair‐associated transcription factors [64]. Our study further found that tumor‐expressed ITGA5 interacts with FN1 in the SC extracellular matrix, triggering reprogramming of SCs into a repair phenotype [37]. Together, these findings suggest that SC activation is likely the result of coordinated stimulation through multiple pathways (Figure 3).

Despite these parallels, tumor‐induced Schwann cell activation does not represent a uniform recapitulation of canonical repair states. Single‐cell and spatial transcriptomic analysis of clinical pancreatic samples identified a distinct TGFBI^+^ SC subpopulation beyond the canonical myelinating and repair subtypes, characterized by ECM remodeling and growth factor expression [13]. Induced by TGF‐β signaling within the TME, this population enhances cancer cell migration and invasion, underscoring the functional heterogeneity of TASCs and extending their role beyond classical injury‐associated repair programs [13].

### 4.2 TASCs‐Mediated Multidimensional Mechanisms Promoting PNI

#### 4.2.1 Physical Guidance and Mechanical Forces Mediated by Direct Contact

Emerging evidence indicates that SCs are not merely passive bystanders in PNI but active pioneers of tumor invasion. Demir et al. reported that activated SCs exhibit tumor‐directed chemotaxis in pancreatic and colon cancer. They detach from nerve bundles and migrate toward tumor cells, establishing direct contact at the invasive front [57]. This active, tumor‐directed recruitment is further corroborated in CCA and colorectal cancer models [44, 66]. This behavior recapitulates the formation of “bands of Büngner” seen in Wallerian degeneration, guiding neurite outgrowth toward tumors while simultaneously creating preformed tracks that enhance the efficiency and directionality of cancer cell migration. Building on this concept, Deborde et al. demonstrated that activated GFAP^+^ SCs dynamically shuttle between nerves and tumors. They infiltrate tumor cell clusters, disrupt intercellular junctions, and reorganize tumor cells into chain‐like structures that favor single‐cell dispersal [56]. Subsequent work showed that c‐Jun–reprogrammed SCs generate linear tumor‐activated Schwann cell tracks (TASTs), along which cancer cells migrate directionally toward nerves [58]. These SCs also exert active mechanical forces—pushing, pulling, and compressing—on tumor cells, further enhancing motility and invasion [58]. To structurally accommodate this physical invasion, reprogrammed SCs acquire a robust capacity for extracellular matrix (ECM) remodeling. By secreting high levels of MMPs (e.g., MMP1) and pro‐angiogenic factors (such as VEGF and FGF2), effectively paving the way for physical tumor expansion, neovascularization, and nerve infiltration [64].

Direct SC–tumor interactions are mediated by specific cell adhesion molecules. Single‐cell transcriptomic analyses link the pro‐PNI activity of TASCs to enhanced adhesion, migration, and axon‐guidance programs [13]. Functionally, neural cell adhesion molecule 1 (NCAM1) is highly expressed in SCs and contributes to cancer cell guidance during PNI. Inhibition of NCAM1 attenuates SC‐driven invasion in experimental models [56]. In PDAC, L1CAM, a canonical regulator of axonal growth and neuronal migration, is aberrantly upregulated in activated SCs, acting as a chemoattractant for cancer cells [67, 68]. Mechanistically, L1CAM can activate STAT3 signaling and induce MMP‐2 and MMP‐9, which is consistent with enhanced neural invasion. In vivo inhibition studies and clinical tissue analyses further support an association between L1CAM and PNI [67]. In addition, myelin‐associated glycoprotein on perineural myelinating SCs binds Mucin 1 on pancreatic cancer cells, forming an adhesive bridge that facilitates tumor cell attachment and penetration into myelinated nerves, thereby promoting the initiation of PNI [69].

#### 4.2.2 Paracrine Signaling and Chemotaxis

Beyond direct contact‐mediated scaffolding, SCs also promote PNI by secreting paracrine factors. These signals form a coordinated chemotactic network that guides cancer cell migration and tumor–nerve interactions [55].

Specifically, SCs establish these chemotactic signaling circuits by secreting multiple neurotrophic factors, including BDNF, NGF, NT‐3, and GDNF. These ligands can stimulate neurite outgrowth toward tumors and, in turn, promote cancer cell migration along neural structures.

NGF promotes PNI by inducing EMT and enhancing cancer cell invasiveness. In addition to tumor cell‐autocrine NGF [52], TASC‐ and neuron‐derived NGF together provide chemotactic cues that guide cancer cell migration [37, 55]. The NGF/p75NTR axis also supports SC migration between tumors and nerves. In pancreatic cancer models, genetic deletion of p75NTR or pharmacologic blockade (e.g., RO.08‐2750) reduces SC migration and suppresses PNI [57].

In parallel, the BDNF/TrkB signaling axis has been identified as a key pathway through which SCs regulate PNI across multiple tumor types. Across various malignancies, SC‐derived BDNF promotes PNI primarily by enhancing tumor cell migration and EMT [70, 71]. In specific contexts, such as salivary adenoid cystic carcinoma (SACC), BDNF/TrkB signaling additionally drives Schwann‐like differentiation of tumor cells, further facilitating neural invasion [72]. Across cancers, activation of this axis enhances tumor cell migration and invasion and promotes the formation of nerve–tumor synapse‐like structures, collectively increasing neural invasiveness [73]. Pharmacologic blockade of TrkB (e.g., ANA‐12, K252a, entrectinib) attenuates these effects, highlighting the central role of this axis in PNI [72, 73]. In addition, interactions mediated by the NT‐3/TrkC axis have also been shown in vitro to promote SACC cell migration, proliferation, and PNI progression [74].

In PDAC and chronic pancreatitis, SCs undergo adaptive reprogramming and upregulate GDNF to repair injured nerves [75]. Consistent with its role in neural repair, GDNF acts as a potent chemoattractant for pancreatic cancer cells and promotes tumor growth and invasion along nerve axons via RET signaling and its downstream PI3K/ERK and MAPK pathways [76, 77, 78], as demonstrated in murine sciatic nerve models. SC‐derived GDNF and related family members, including artemin and neurturin, promote PNI in PDAC, with expression levels correlating with neural invasion severity in patients [79].

Beyond neurotrophic factors, SC‐derived CXCL5 has been shown in lung cancer to promote EMT and tumor cell motility, thereby enhancing invasiveness and metastatic potential [80]. Furthermore, emerging evidence highlights metabolic crosstalk as another crucial driver of SC activation. Pascual et al. revealed that, in oral carcinomas and melanoma, dietary palmitic acid induces a “neural‐like” transcriptional state in tumor cells, which in turn activates TASCs to acquire a pro‐regenerative phenotype. These TASCs secrete a regenerative ECM that not only promotes migration and neural invasion but also serves as a central mechanism for the formation of metastatic memory [81]. Similarly, in distal CCA, tumor‐derived lactate within the hypoxic microenvironment induces dedifferentiation of Schwann cells into a GFAP^+^ state, which promotes cancer cell invasion and PNI through HMGB1 upregulation, further highlighting a metabolically driven mechanism of SC activation [82].

Notably, SCs exhibit significant heterogeneity in modulating tumor‐nerve interactions. Different SC subtypes may exert divergent effects on PNI. Sroka et al. demonstrated that myelinating SCs (S16) secrete soluble factors inducing ITGA6 expression in tumor cells, enhancing integrin‐dependent migration and neural invasion in prostate cancer cells. Conversely, non‐myelinating SCs (S16Y) inhibit invasion [83]. These subtype‐specific functions within the tumor microenvironment reveal the complexity of PNI regulation, suggesting that future therapeutic strategies must precisely discriminate among SC subpopulations.

Interestingly, Cancer‐induced SC activation has been associated with modulation of nociception, as activated SCs upregulate anti‐inflammatory cytokines while downregulating pain‐related gene programs [62]. These findings suggest that cancer‐induced SC activation not only remodels the neural niche but may also mask pain signals during tumor progression, thereby creating a permissive, immunosuppressive milieu favorable for tumor growth, although context‐dependent pro‐nociceptive effects have also been reported [84].

Overall, the behaviors of SCs during tumor progression closely mirror their activation programs during nerve repair. However, the precise regulatory mechanisms governing these processes remain incompletely understood. Future studies should focus on dissecting SC subtype‐specific functions and their crosstalk with other stromal and neural components to identify novel targeted strategies for intercepting neural invasion.

## 5 The Impact of the Immune Microenvironment on PNI

During PNI, the immune microenvironment constitutes a critical component of the tumor–nerve interface. By orchestrating local inflammation and remodeling both stromal and neural architectures, immune cells and their secretomes profoundly influence the behavior of cancer cells and SCs. Accumulating evidence suggests that multiple immune cell populations may facilitate neural invasion by releasing neurotrophic factors and immunomodulatory cytokines. Conversely, certain immune cell subsets and immune‐associated structures may exert inhibitory effects on PNI by eliciting anti‐tumor inflammatory responses. Collectively, these findings underscore the immune microenvironment as an integral and dynamic participant in PNI progression [85].

### 5.1 Tumor‐Associated Macrophages Orchestrate Perineural Invasion

As the dominant immune population within the tumor stroma, tumor‐associated macrophages (TAMs) fundamentally drive tumor progression through matrix metalloproteinase‐mediated ECM remodeling and the canonical EGF/CSF‐1 paracrine loop, creating permissive routes for tumor invasion [86]. Crucially, this intrinsic matrix‐remodeling capacity shares mechanistic commonalities with the breaching of neural barriers in PNI. Multiple clinical studies in pancreatic cancer have demonstrated a significant positive correlation between TAM infiltration, particularly the co‐localization of CD68^+^ macrophages with GFAP^+^ SCs, and PNI occurrence [13, 56, 87, 88].

Macrophages exert dual roles within the PNS: M1 phenotypes mediate inflammation and injury, while M2 phenotypes release neurotrophic factors to support neuronal migration, neurogenesis, and nerve regeneration. This functional dichotomy is recapitulated in the tumor‐associated neural microenvironment. Given that the TME preferentially polarized macrophages toward an M2 phenotype [89], PNI regions are often enriched with these immunosuppressive, pro‐neurogenic M2‐TAMs [90]. Within this niche, the interaction evolves into a dynamic SC‐TAM feedforward loop. In CCA, Wang et al. revealed that cancer cell‐derived exosomes drive M2 polarization and robust CCL2 secretion, promoting directional tumor migration while concurrently activating neurons and SCs [91]. Similarly, in colorectal cancer, the JAG2–NOTCH3 axis similarly promotes PNI by inducing STAT3‐dependent CCL2 expression in M2‐polarized TAMs [92]. Once polarized, M2‐TAMs secrete neurotrophic factors—specifically promoting SC migration and proliferation via the bFGF/PI3K/Akt signaling pathway—to accelerate neural tract remodeling and facilitate PNI progression [87]. Reciprocally, reprogrammed SCs act as active drivers of this immunosuppressive niche. In response to tumor‐induced injury or paracrine cues, TASCs orchestrate a complex pro‐healing network by secreting a diverse array of immunomodulatory factors, including IL‐6, MIF, TGF‐β1, Galectin‐3, and IL‐33 [64, 87]. This robust paracrine signaling not only recruits macrophages to the invasive front but decisively drives their M2 polarization, thereby forming a potential SC‐TAM feedforward loop that amplifies pro‐PNI signaling. In parallel, TAM‐derived IL‐1β and TGF‐β cooperate with SCs to further sculpt a pro‐invasive microenvironment, thereby amplifying pro‐PNI signaling and accelerating PNI initiation and progression [13].

Beyond signaling amplification, TAMs execute the physical breaching of nerve sheaths. In a study of PDAC and prostate cancers by Bakst et al., macrophages recruited via the CCL2‐CCR2 axis differentiate into a specialized subset characterized by high expression of Cathepsin B (CTSB). Elevated CTSB degrades collagen IV within the perineurium, facilitating the breach of the neural barrier and subsequent tumor cell invasion [93]. The CCL2 axis exhibits multi‐source regulation within the tumor microenvironment. In addition, M2 macrophages can form low‐immune‐pressure niches surrounding nerve bundles, further accelerating the initiation and expansion of PNI [91].

Collectively, M2‐polarized TAMs exert multi‐layered regulation in PNI by promoting tumor invasion, driving SC activation and neural remodeling, participating in nerve‐tumor‐immune feed‐forward loops, and directly disrupting neural barrier structures.

Of note, the PNS harbors resident immune cells known as endoneurial macrophages (EMΦ), which, although distinct from conventional TAMs, also play critical roles in PNI. Cavel et al. observed a marked increase in EMΦ density around nerves invaded by cancer. Using DRG co‐culture models combined with video microscopy and time‐lapse analysis, they demonstrated that EMΦ actively migrate toward the tumor front and facilitate tumor invasion into DRGs [94]. Further mechanistic studies revealed that tumor‐activated EMΦ (tEMΦ) secrete elevated levels of GDNF, inducing RET phosphorylation and downstream ERK activation in PDAC cells, thereby enhancing tumor cell migration and invasion and ultimately promoting neural invasion [94].

### 5.2 Non‐Macrophage Immunity in PNI: Emerging Evidence and Challenges

While accumulating studies have highlighted the intricate crosstalk among the tumor‐nerve‐immune axis, current evidence primarily focuses on delineating the consequences of PNI on immune remodeling: PNI zones typically exhibit a distinct immunosuppressive landscape characterized by the exclusion of anti‐tumor effectors (CD4+/CD8+ T cells, dendritic cells, NK cells) and the recruitment of suppressive populations (MDSCs, Tregs) [10]. However, it remains largely undetermined whether these non‐macrophage immune subsets actively initiate PNI or merely respond to established neuroinflammatory cues. Clinical observations have identified certain immune populations, such as mast cells, enriched in perineural regions; however, their primary role appears limited to modulating cancer‐associated pain rather than directly driving neural invasion [95]. While neurons are intrinsically sensitive to immune‐derived inflammatory signals [96], the specific soluble factors that drive bidirectional tumor–nerve interactions during PNI remain poorly defined. Similarly, the role of tertiary lymphoid structures (TLSs) in PNI is currently supported primarily by correlative evidence. In pancreatic cancer, nerve‐TLS structures (NTSs) have been observed preferentially in non‐invaded regions, whereas they are markedly depleted in PNI‐positive areas [13]. Similar correlations in breast cancer suggest that TLSs might establish a locally protective environment that constrains neural invasion [97]. Nonetheless, whether TLSs provide a functional barrier to invasion or simply reflect a robust anti‐tumor response in less aggressive regions remains to be determined. Future research utilizing spatial transcriptomics and tumor‐specific models will be essential to transition from these correlative observations to definitive mechanistic insights, delineating whether non‐macrophage immune cells act as active regulators or passive responders within the tumor–nerve–immune axis, thereby informing potential therapeutic strategies targeting the tumor–nerve–immune axis.

## 6 A Neuro‐Ecological Model of the Perineural Niche

By integrating diverse stromal components and their reciprocal interactions, PNI can be conceptualized as a dynamically evolving, multicellular ecosystem. Within this framework, the progression of PNI cannot be fully explained by isolated signaling events alone, but instead reflects the integration of multiple interacting dimensions.

### 6.1 Stroma‐Mediated Niche Construction and Structural Remodeling

Stromal components, most notably CAFs, SCs, and specific immune populations, actively remodel the perineural environment. Through the secretion of matrix‐degrading enzymes (e.g., MMPs, CTSB) [33, 93] and the deposition of aligned ECM fibers, these cells transform the restrictive perineurial barrier into a structurally and biochemically permissive conduit for neural invasion.

### 6.2 Feed‐Forward Amplification via Paracrine

The maturation of the perineural niche involves reciprocal feedback interactions not only between tumor cells and the surrounding microenvironment, but also among stromal components themselves. This feed‐forward amplification appears to arise from bidirectional paracrine coupling across multiple cellular compartments. Within the CAF–SC axis, CAFs drive Schwann cells toward pro‐invasive and demyelinating states, while Schwann cells in turn promote inflammatory CAF polarization. Together, these reciprocal interactions create a self‐reinforcing cycle of nerve injury and structural remodeling. This network is further amplified by SC–macrophage coupling, where sustained paracrine exchanges maintain M2‐macrophage polarization and accelerate SC migration. In addition, these inter‐stromal interactions directly influence the local immune landscape [10, 98]. By suppressing cytotoxic responses and facilitating the recruitment of specific immunosuppressive populations, these intercellular feedback loops collectively sustain a pro‐invasive microenvironment. Although the primary focus of this Review is stromal regulation of PNI, neural‐intrinsic pathways likely represent an important parallel layer of perineural niche biology.

### 6.3 Heterogeneity and Spatial Compartmentalization

Building upon the reciprocal signaling networks described above, the perineural niche is spatially organized into a highly compartmentalized ecosystem where distinct stromal cell states execute non‐redundant functions based on their topographical positioning (Table 1). At the tumor–nerve invasive front, pro‐invasive populations—such as matrix‐remodeling myCAFs and reprogrammed Schwann cells—closely co‐localize. This spatial proximity facilitates the paracrine crosstalk, enabling targeted barrier degradation and the formation of physical guidance tracks. Within this spatial hierarchy, structural parameters such as the diameter of the involved nerves and tumor–nerve distance may act as key determinants of niche organization. Specifically, nerve diameter influences the physical interface available for stromal–tumor engagement, while tumor–nerve distance may define gradients of chemotactic signaling and the spatial extent of tumor–nerve interactions. These features have also been associated with disease aggressiveness in clinical settings, potentially reflecting the maturity of the perineural niche [20]. Beyond this immediate interface, peripheral supportive niches harbor functionally specialized subsets, such as tCAFs providing localized metabolic support or iCAFs orchestrating immune suppression. Conversely, spatially distant or structurally intact regions retain baseline or restrictive populations (e.g., progenitor‐like CAFs and non‐myelinating SCs) that do not actively participate in tumor invasion and PNI. Ultimately, this spatial compartmentalization not only coordinates the mechanical, metabolic, and immunological demands of PNI but also establishes the structural basis for its stage‐wise ecological progression.

**TABLE 1:** Key Fibroblastic and Schwann Cell States in Perineural Niches. Ecological functional classification is applied to integrate distinct subpopulations based on their specialized roles within the perineural ecosystem. Subtype/state annotations and representative markers were retained as reported in the original studies, or indicated as undefined (‐) when lacking specific outputs in the context of PNI.

Stromal population	Ecological Functional Class	Subtype / state	Representative markers	Spatial localization	Specific Contribution to PNI		Cancer context	References
					Outputs	Functional effect		
CAFs	Pro‐invasive & Matrix‐Remodeling	**Myofibroblastic CAFs (myCAFs)**	FAP^^^hi, α‐SMA^^+^, CD138^^+^, MMP‐11^^+^, TGF‐β‐enriched state	Enriched in high‐PNI tissues; invasive edge of nerves	ECM, MMPs, TGF‐β	ECM remodeling; pro‐invasive stromal signaling; induction of pro‐invasive Schwann‐cell states	PDAC	[13]
		**Perineural CAFs / MMP‐2^+^ CAF state**	MMP‐2^^+^	Perineural regions; absent or low in non‐PNI regions	MMP‐2	ECM degradation; weakens local barrier integrity and facilitates early neural invasion	OSCC	[33]
		**Adhesive / Matrix‐Remodeling CAFs states**	FAP, Nectin‐1	Tumor–stroma–nerve interface	Integrin‐ligands, adhesion molecules (e.g., Nectin‐1)	Strengthens tumor–nerve adhesion and promotes neurotropic invasion	PDAC, cSCC	[35, 36, 38]
		**Pancreatic stellate cells (PSC‐like fibroblast subset)**	α‐SMA^^+^, COL1A1^^+^, FN1^^+^ (largely overlapping with myCAFs)	PDAC stroma / perineural niche	NGF and MMP9 upregulation via HGF/c‐Met/mTOR; SHH signaling; tenascin C production	Promotes PNI and bidirectional tumor–nerve tropism	PDAC	[51, 52, 53, 54]
	Immunomodulatory	**Inflammatory CAFs (iCAFs)**	CD34^^+^, IL‐6^^hi^, CXCL12^^hi^	Perineural niches proximal to Schwann cells	Pro‐inflammatory cytokines (IL‐6, LIF), Chemokines (CXCL12, CCL2)	Promotes tumor cell migration and orchestrates a localized immunosuppressive niche	PDAC	[50]
	Metabolic & Angiogenic Support	**Tumor‐like CAFs (tCAFs)**	MME^^+^, NDGR1^^+^, ENO1^^+^, VEGFA^^+^	Enriched in PNI‐high tissues; proximity to hypoxic regions	VEGFA; hypoxia pathway mediators	Associated with high‐PNI stromal architecture; associated with chemoresistance	PDAC; Breast cancer	[13, 99]
	Base line &Spatially Distant	**Progenitor‐like CAFs**	PI16^^+^, FAP^^lo^, DPT^^+^, CFD^^+^	Enriched in non‐PNI tissues	—	—	PDAC	[13]
Schwann cells	Homeostatic & Physiological States	**Myelinating Schwann cells**	MAG^^+^, MBP^^+^, GalC^^+^	Myelinated/perineural nerve compartments	MAG, soluble factors	Promotes early tumor‐nerve adhesion via the MAG‐MUC1 axis and secretes factors inducing invasion along neural laminin.	PDAC, Prostate cancer^a^	[69, 83]
		**Repair Schwann cells (rSCs)**	c‐Jun^^+^, GFAP^^^+, SOX2^^+^, p75NTR^^+^	Injured/regenerating nerves; repair tracks (“bands of Büngner”)	Neurotrophic factor secretion; axon‐guiding structures; macrophage recruitment	Provides the physiological repair program that can be hijacked during PNI	General PNS repair biology used as conceptual template for PNI	[59]
	Reprogrammed & Pro‐invasive	**Tumor‐activated Schwann cells (TASCs) / Repair‐like state**		Tumor invasive front; between nerves and tumor cell clusters	Track formation; mechanical guidance; neurotrophic secretion; ECM remodeling	Actively guides tumor‐cell migration toward nerves and amplifies PNI	PDAC, Melanoma, HNSCC	[37, 58, 64]
		**TGFBI^+^ Schwann‐cell state**	TGFBI^^+^, FN1^^+^	Invading edge of nerves; direct contact interface with tumor cells and adjacent to TGF‐β1^^+^ myCAFs/TAMs	TGFBI, FN1, ECM, growth factors.	Enhances cancer‐cell migration and invasion within the perineural niche	PDAC	[13]
	Restrictive States	**Non‐myelinating Schwann cells**	MAG^^−^, GalC^^^−, P0^^−^, S100β^^+^	Non‐myelinated nerve compartments	Less permissive or inhibitory secretory influence in specific models	May oppose or reduce invasion relative to myelinating Schwann cells	PDAC ^a^, Prostate cancer ^a^	[83]

^a^ Evidence primarily derived from in vitro co‐culture models.

### 6.4 Dynamic Stromal States across the Stages of PNI

**FIGURE 4:**
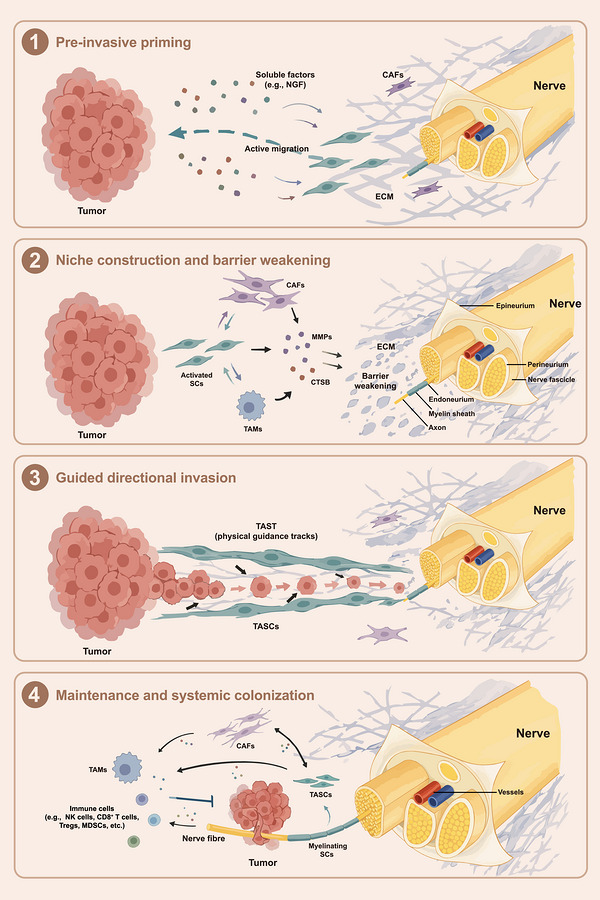
Dynamic stromal states across the four stages of PNI. (1) Pre‐invasive priming: Tumor‐derived soluble factors (e.g., NGF) stimulate the active migration of Schwann cells (SCs) toward the tumor. (2) Niche construction and barrier weakening: CAFs, activated SCs, and TAMs secrete proteases (MMPs, CTSB) to degrade the extracellular matrix and compromise the nerve sheath barrier. (3) Guided directional invasion: Tumor‐associated SCs (TASCs) establish physical guidance tracks (TASTs) that mechanically facilitate directional tumor cell invasion. (4) Maintenance and systemic colonization: TASCs and TAMs orchestrate a robust immunosuppressive microenvironment, inhibiting cytotoxic immune cells (e.g., NK and T cells) to protect colonizing tumor cells.

PNI progression may follow a staged, but potentially overlapping, ecological succession, in which cellular division of labor appears to be coordinated across expanding anatomical domains. This process can be broadly conceptualized as comprising four stages (Figure 4):

**Stage 1: Pre‐invasive priming**. The succession initiates in the extra‐neural environment, where tumor‐derived soluble factors (e.g., NGF) long‐range modulate the neural microenvironment. During this phase, SCs sense early chemotactic gradients and begin their active migration toward the tumor front, acting as pioneering navigators before direct contact.
**Stage 2: Niche construction and barrier weakening**. As the invasive front reaches the perineurial interface, CAFs and activated SCs secrete MMPs, complemented by CTSB release from TAMs. Together, this stromal consortium reorganizes the perineurial ECM and compromises the integrity of the nerve sheath, effectively converting a restrictive physical barrier into a permissive gateway.
**Stage 3: Guided directional invasion**. Coupled with localized barrier remodeling, the invasive process relies heavily on active directional guidance. TASCs occupy the leading edge, forming physical guidance tracks (TASTs) that provide directional migratory support and mechanical force to tumor cells, facilitating their rapid dispersal along and within the neural architecture.
**Stage 4: Maintenance and systemic colonization**. The succession culminates in a stabilized colonization phase within the neural architecture. Once tumor cells establish residency within the nerve, accumulating evidence highlights a profound transition toward neuro‐immune suppression. Driven by extensive tumor–nerve–immune crosstalk, metabolically reprogrammed TASCs and M2‐polarized TAMs collaboratively orchestrate a robust immune evasion network. Within the local niche, the secretion of immunomodulatory factors directly paralyzes cytotoxic effectors, including T cells and NK cells, establishing a persistent, immune‐privileged shield [10, 37]. Furthermore, this neuro‐immune axis can even exploit neural circuitry to mediate immunosuppression within distal tumor‐draining lymph nodes [90]. This comprehensive immune evasion protects the colonizing tumor cells from clearance, ensuring the long‐term survival and recurrence of the neuro‐invasive ecosystem.

The cellular logic of PNI appears to follow a staged progression, transitioning from pre‐invasive priming and barrier weakening to guided invasion along nerves and the subsequent maintenance of a pro‐neuroinvasive state. These spatial and temporal features are likely interdependent, collectively contributing to the functional organization and persistence of the perineural niche.

Crucially, this neuro‐ecological framework provides mechanistic clarity to the profound resilience of PNI in clinical settings. The high stability and irreversibility of PNI may be partly explained by the presence of feed‐forward amplification loops, which could enable sustained interactions within the perineural niche even in the context of fluctuating tumor‐derived signals. The physical guidance provided by neural structures, together with sustained immunosuppression and trophic support within the niche, may further facilitate tumor dissemination, including local progression and lymphatic or distant metastasis [100, 101]. Furthermore, the inherent spatial compartmentalization and functional redundancy among niche components, such as parallel chemotactic support provided by both CAFs and TASCs, explain why single‐target therapies (e.g., blocking a single neurotrophic receptor) frequently fail in PNI‐positive malignancies [44]. Recognizing PNI as an emergent ecological network rather than a linear pathway underscores the necessity of paradigm‐shifting therapeutic strategies, which will be discussed in the subsequent section.

## 7 Therapeutic Opportunities and Translational Challenges Across the Stages of Perineural Invasion

Therapeutic opportunities in PNI are likely to be stage‐informed rather than pathway‐agnostic, and treatment selection may be most reasonably guided by a clinicopathologic estimate of PNI stage rather than a formal staging system. Within this framework, earlier PNI stages 1–3 are likely to be clinically occult in many patients and therefore inferred mainly from pathology, imaging, and tumor context. By contrast, stage 4 more commonly coincides with clinical evidence of established neural invasion, including pain, numbness, paresthesia, or functional deficits. In the priming stage, strategies that attenuate stress and inflammatory signaling may be most relevant, as illustrated by the phase II randomized PROSPER trial of perioperative propranolol plus etodolac in pancreatic surgery, which showed acceptable safety but primarily exploratory efficacy signals [102]. During niche construction and barrier loosening, strategies targeting dense stromal ECM may be particularly relevant, as illustrated by the randomized phase II HALO 202 trial of PEGPH20 plus nab‐paclitaxel/gemcitabine in metastatic PDAC [103]. In active neural invasion, blockade of neurotrophic guidance pathways is conceptually supported by the phase 1/2 trial of repotrectinib in NTRK fusion‐positive solid tumors, which demonstrated durable activity across tumor types [104]. In the maintenance and systemic colonization stage, treatment may need to prioritize the neuro‐immune axis, particularly macrophage‐directed and adrenergic circuits, as illustrated by the phase I/II study of BMS‐813160 plus nivolumab and chemotherapy in pancreatic cancer and by emerging evidence implicating β2‐adrenergic macrophage signaling in sustained immune escape [105]. Overall, these findings support a stage‐matched therapeutic framework for PNI, in which clinicopathologic assessment guides selective targeting of the dominant stromal components at each phase of disease evolution.

## 8 Limitation and Future Direction

Despite these stage‐matched therapeutic opportunities, the neuro‐ecological model of PNI should still be regarded as a working framework rather than a fully resolved biological architecture. Although direct evidence for the participation of vascular components (e.g., endothelial cells and pericytes) in PNI is still lacking, neurovascular coupling and shared signaling pathways suggest a potential role in shaping the perineural niche. However, these interactions remain to be validated in tumor‐specific contexts. Similarly, immune regulation beyond macrophages remains comparatively underexplored. CAFs, Schwann cells, and immune cells likely occupy distinct and shifting functional positions across stages of neural invasion, but most available studies still rely on endpoint pathology, bulk associations, or reductionist co‐culture systems, limiting inference of causal hierarchy and reciprocal state transitions. Future progress will therefore require cross‐cancer comparative studies, integrated single‐cell and spatial analyses, lineage‐aware in vivo models, and neuro‐tumor organoid systems that capture the staged evolution of the perineural niche. Clinically, the major challenge is that many candidate targets overlap with physiological nerve repair and immune homeostasis. The next advance in the field will depend on identifying stage‐selective biomarkers and context‐specific vulnerabilities that convert this conceptual framework into a clinically actionable model.

## Author Contributions

X.L., Y.L., and X.L.: conceptualization. X.L., Y.L., H.L., and K.F.: data curation. X.L. and Y.L.: writing – original draft. X.L.: visualization. All authors: Writing – review & editing.

## Funding

This work was supported by the National Natural Science Foundation of China (82172752, 82573154).

## Ethics Statement

Ethics approval was not required for this review article, as it did not involve human participants, human tissues, animal experiments, or identifiable private information.

## Consent for Publication

No written consent for publication was required, as this review article does not contain any patient‐identifiable data.

## Trial Registration

Not applicable. This review article is not a clinical trial.

## Conflicts of Interest

The authors declare no conflicts of interest.

## Data Availability

Data sharing not applicable to this article as no datasets were generated or analysed during the current study.
